# “Men Rule… this is the Normal Thing. We Normalise it and it’s Wrong”: Gendered Power in Decision-Making Around Sex and Condom Use in Heterosexual Relationships Amongst Adolescents and Young People in South Africa

**DOI:** 10.1007/s10461-022-03935-8

**Published:** 2022-11-28

**Authors:** Zoe Duby, Kate Bergh, Kim Jonas, Tarylee Reddy, Brittany Bunce, Chantal Fowler, Catherine Mathews

**Affiliations:** 1 Health Systems Research Unit, South African Medical Research Council, Cape Town, South Africa; 2 Division of Social and Behavioural Sciences in the School of Public Health and Family Medicine, University of Cape Town, Cape Town, South Africa; 3 Biostatistics Research Unit, South African Medical Research Council, Cape Town, South Africa; 4 Institute for Global Sustainable Development (IGSD), University of Sheffield, Sheffield, UK

**Keywords:** Sexual Relationship power, Sex, Condom use, Sexual decision-making, South Africa, Adolescents and Young people

## Abstract

We examined power and decision-making in heterosexual relationships amongst South African adolescents and young people. A survey conducted with 515 adolescent girls and young women (AGYW) included items from the Sexual Relationship Power Scale (SRPS) adapted for South African women. Qualitative interviews with fifty AGYW aged between 15 and 24, and nine males aged 18 years and above, explored decision-making in heterosexual relationships, particularly relating to timing of sex and condom use. Theories of gendered power, sexual relationship power and sexual scripting were used in interpreting the data. Findings showed that the power AGYW have in sexual relationships determines their ability to use condoms, and that males generally control condom use and timing of sex. Both survey and interview data suggest that male control over female partners’ behaviour also extends beyond the sexual domain. Although while male power is pervasive and enduring, it is simultaneously contested and negotiated. Despite some young people believing that gendered power in decision-making should be equal, it is not always possible for AGYW to enact agency in the dyadic context of heterosexual relationships. Whilst adolescents and young people in South Africa move away from traditional cultural gendered expectations, relationship power inequity and hegemonic masculinities continue to legitimise men’s power over women, constraining the sexual agency of adolescent girls and young women and discouraging them from taking control of their own sexual interests and sexual health.

## Introduction

Women’s ability to exert agency in decision-making and negotiation of timing of sex and condom use are key determinants of sexual and reproductive health (SRH) outcomes [1]. Recent studies and social theorists have built upon the theory of Gender and Power [2], recognising the persistence of structurally embedded power differentials in heterosexual sexual and romantic relationships, which determine decision-making around the use of contraceptives and condoms, and the timing of sex [1, 3, 4]. Relationship power consists of two dual components: “power to” (the ability to act as one desires), and “power over” (the ability to assert desires, even in the face of opposition), and manifests in the ability of one partner to dominate decision-making in the relationship [5, 6].

Sexual relationship power refers to the levels of influence partners have over decision-making in the relationship context, and the extent to which one partner has control over their partner’s actions [7]. Power inequities in sexual and romantic relationships between men and women traditionally manifest in male control over decisions around timing of sex and safe sex practices [8]. Gendered power inequities in sexual relationships tend to be associated with poor SRH outcomes for women globally, as well as with numerous determinants of HIV acquisition, including condom use, highlighting potential pathways from SRP inequity to HIV risk of adolescent girls and young women (AGYW) [3, 4, 9, 10]. Sexual relationship power has been measured in studies using instruments such as the Sexual Relationship Power Scale (SRPS), developed and validated more than two decades ago by Pulerwitz et al. [11] in order to address the need to measure relationship power in intimate and sexual relationships, enabling researchers to examine the effects of power differentials on sexual health outcomes for women [3, 12].

In the context of condom use behaviour, although the majority of evidence suggests that women who report lower relationship power are more likely to report inconsistent condom use, studies conducted in various parts of the world, most notably in the United States, have found mixed evidence on the correlations between sexual relationship power and condom use [7, 9, 13, 14]. Similar contrary evidence has emerged in the South African context, where there have been questions raised as to whether SRP equity is associated with condom use [8, 15]. These studies demonstrate limitations in the SRPS, given that it asks specifically about respondents’ primary partners, and thus fails to capture different forms of sexual relationships [8].

Noting this limitation, and considering that sexual behaviour occurs within social and dyadic contexts, we incorporated the Sexual Scripting theory into our analysis. The Sexual Scripting theory seeks to provide a framework for understanding sexual behaviour in terms of socio-cultural norms and guidelines for how partners should behave within sexual relationships [1, 16]. Gendered power dynamics and decision-making are central to sexual interaction, which makes the scripting theory useful in examining relationship contexts and gendered power dynamics that influence an individual’s ability to engage in safe sex practices [16, 17]. Gendered sexual scripts determine the extent to which partners are able to exert power in the dyadic context, and their agency in decision-making around sex and condom use [16]. It has been suggested that within the context of sub-Saharan Africa, sexual behaviour and decision-making processes are more influenced by socio-cultural norms than by individual-level factors [18]. Therefore, using the sexual scripting theory is helpful in this context.

Given the contextual specificities of sexual relationship dynamics, and the way in which gendered power and sexual scripts are informed by and embedded within specific sociocultural and temporal settings, we aimed to examine the complex dynamics of gendered power in decision-making relating to sex and condom use within heterosexual relationships amongst adolescents and young people in South African communities characterised by high HIV prevalence and teenage pregnancy. For our analysis, we used the frameworks of the Gender and Power theory, and Sexual Relationship Power, combined with aspects of the Sexual Scripting theory to interpret data from a survey with AGYW, and qualitative interviews with AGYW and male peer respondents.

## Methods

We analysed data from a cross-sectional telephone survey and remotely conducted qualitative interviews, that were nested within a study evaluating an intervention for AGYW in South Africa^1^. Data collection took place between November 2020 and March 2021, in six districts, spanning six provinces of South Africa: Klipfontein (Western Cape), King Cetshwayo (KwaZulu Natal), Ehlanzeni (Mpumalanga), Bojanala (North West), Nelson Mandela Bay (Eastern Cape), and Thabo Mofutsanyana/Dihlabeng (Free State).

### Survey Component Methodology

Between December 2020 and February 2021, we conducted a cross-sectional telephone survey^2^ with the aim of surveying 1260 AGYW who had participated in the intervention. However, 515 were contactable by telephone and consented to participate; a sample realization of 23.8%. Additional details on sampling have been published elsewhere^2^.

We included measures from the Sexual Relationship Power Scale (SRPS). The original SPRS was a 23-item theoretically based and validated measure of relationship power dynamics, comprising two subscales concerning conceptual dimensions of relationship power which can be used separately or combined: Relationship Control and Decision-Making Dominance. Items on the Relationship Control subscale are scored on a 4-point Likert scale (1 = strongly agree to 4 = strongly disagree) [9]. A modified version of the SRPS, the SRPS-M, eliminates items related to condom use so that the scale can be used to more accurately predict safer sex behaviours [11]. Different versions of the SRPS have been widely used in research examining gender inequities in heterosexual relationships in sub-Saharan Africa [3]. In 2002, Jewkes et al. [12] developed an adapted 13-item version of the SRPS specifically to measure sexual relationship power equity amongst adolescents in the South African context [8].

In our analysis, we included measures from the version of the SRPS adapted for South African women, and potential confounders as independent variables, with condom use as the dependent variable. Measures from the SRPS included a set of eight statements about the participant’s relationship with her current or most recent main partner/boyfriend, and she was asked the extent to which she agreed, with response options ranging from strongly agree through to strongly disagree. If she was not sure, or if she had not been in the situation, we asked her to imagine how he would act. Potential confounders included age, socio-economic status, having ever been pregnant, HIV status, relationship status, age-disparate sexual relationships and use of contraceptives other than condoms in the past six months. In terms of condom use, participants were asked what percentage of the time they had used condoms while having sex with the last boy or man they had sex with.

AGYW were asked to give an estimate of the percentage of time they used condoms when having sex with their last partner. We divided condom use reporting into tertiles: used condoms 0–49% of the time, 50–75% of the time, and 76–100% of the time. In addition, we present survey data on AGYW reporting of reasons why they had not used condoms 100% of the time during sex in the last 3 months.

Analysis of survey data was conducted using Stata (StataMP 14, StataCorp, Texas, USA) and R (version 4.0.2). We described key variables with frequencies (n) and proportions (%), overall and stratified by age group. For the multivariate analyses, a multinomial regression model was used to test the association between measures of the SRPS and condom use. This modelling approach was selected due to violation of the proportional odds assumption in ordinal regression models. A multivariate model was built for the measures of the SRPS which had a statistically significant association with condom use when adjusting for potential confounders through an iterative series of bivariate analyses (significance level = 0.05). A variable was considered a confounder and included in this model if it altered the main effect of the SRPS measure on condom use by 10% or more in bivariate analyses. We report relative risk ratios. Missing observations were excluded from the analysis.

### Qualitative Component Methodology

In the qualitative component, we conducted in-depth interviews (IDIs) with fifty (50) AGYW between the ages of 15 and 24 years, and 9 male partner/peer respondents aged 18 years and above. The AGYW who consented for male partners/peers to be contacted, provided phone numbers for the male partner/peer, who was then contacted and invited to participate. Interviews were conducted telephonically, and audio-recorded with participants’ knowledge and consent. Semi-structured interview guides framed discussions, outlining key topics for discussion. Audio recordings were directly translated from their original language into English and reviewed by interviewer/s for accuracy. Qualitative data were coded using iterative thematic analysis, following an integrated and cyclical process using a set of pre-determined deductive code types, reflexively refined to reflect emerging topics during preliminary analysis. Using a process of collaborative interpretation, research team members engaged in data immersion, re-examining data at different stages in the process, documenting reflective thoughts and sharing growing insights during regular discussions. The use of analytic memos created an important extra level of narrative, providing an interface between participants’ data, researchers’ interpretations, and wider theory.

#### Ethical Considerations

Ethical approval to conduct this study was granted by the SAMRC Research Ethics Committee (EC036-9/2020). A study team member contacted each of the participants telephonically to invite them to participate in the study and administered the consent process. For AGYW under 18 years of age, parental/caregiver consent was obtained prior to conducting the consent process with the AGYW. The telephonic consenting process involved the interviewer reading the consent information sheet to the participant in their language of choice, provided in a way that was easy to understand and appropriate to the participants’ education level. Participants were given the opportunity to ask any questions regarding participation. Consent was audio-recorded, and saved securely with the unique participant identification number. Participants were made aware that the consent process and interviews would be audio-recorded. Participants who agreed to participate and gave consent were surveyed or interviewed in their language of choice by a trained interviewer fluent in the local site languages. Each participant received ZAR 100.00 (US$ 7.00) reimbursement for their time taken to participate in the study. To mitigate one possible source of social harms, the study team worked with those AGYW participants who gave permission to us to contact their male partners/peers to carefully explain the potential consequences of allowing their male partners to be contacted for participation (e.g., disclosure of study involvement and/or intervention participation) prior to contacting the male partners.

## Findings

### Survey Findings

**Table 1:** Characteristics of AGYW who participated in the HERStory 2 survey (N=515)

Characteristics	Total	15-19 years old	20-24 years old
	**N=515**	** N=264**	** N=251**
	**Freq (%)**	**Freq (%)**	**Freq (%)**
Age group			
15-19	264 (51.3)	264 (100.0)	0 (0.0)
20-24	251 (48.7)	0 (0.0)	251 (100.0)
Born in South Africa	508 (98.6)	260 (98.5)	248 (98.8)
District (province)			
Klipfontein (Western Cape)	58 (11.3)	15 (5.7)	43 (17.1)
Bonjanala (North West)	63 (12.2)	33 (12.5)	30 (12.0)
King Cetshwayo (KwaZulu-Natal)	126 (24.5)	58 (22.0)	68 (27.1)
Ehlanzeni (Mpumalanga)	108 (21.0)	80 (30.3)	28 (11.2)
Nelson Mandela Bay (Eastern Cape)	70 (13.6)	35 (13.3)	35 (13.9)
Thabo Mofutsanyana (Free State)	90 (17.5)	43 (16.3)	47 (18.7)
Relationship status			
Single	200 (39.0)	138 (52.7)	62 (24.7)
Dating	302 (58.9)	123 (46.9)	179 (71.3)
Living together but not married	10 (1.9)	1 (0.4)	9 (3.6)
Other	1 (0.2)	0 (0.0)	1 (0.4)
Ever had sex	389 (76.3)	156 (59.5)	233 (94.0)
Ever been pregnant	159 (30.9)	40 (15.2)	119 (47.4)
Self-reported living with HIV	15 (3.0)	9 (3.5)	6 (2.4)
Used a contraceptive method other than condoms 100% of the time in the past six months	121 (24.2)	36 (14.1)	85 (34.8)

*There were 1 to 14 missing observations per variable

Of the 515 AGYW survey participants, 264 were in the 15–19 age group, and 251 were in the 20–24 age group. As shown in Table 1, almost all (98.6%) survey participants were born in South Africa. The majority (71.3%) of AGYW aged 20–24 years reported their relationship status as ‘dating’. The majority (52.7%) of AGYW aged 15–19 years reported their relationship status as ‘single’. Most AGYW reported that they had ever had sex (76.3%); 94.0% reported ever having sex in the 20–24 age group, and 59.5% in the 15–19 age group. Almost half (47.4%) of AGYW in the 20–24 years age group reported that they had ever been pregnant; with 15.2% reporting ever having been pregnant in the 15–19 years age group. Overall, 3.0% of AGYW aged 15–24 self-reported to be living with HIV. AGYW self-reporting of having used a contraceptive method other than condoms in the past six months was 14.1% among those aged 15–19, and 34.8% among those aged 20–24 years.

As shown in Table 2, 42.9% of AGYW said that when their partner wants to have sex, they are expected to agree to it. Although most (86.0%) AGYW disagreed that their partner would get angry if they asked him to use a condom, of those AGYW who said their partner would get angry (14.0%), there were more AGYW in the lowest condom use group (54.7%), compared to the higher condom use groups (15.1%). The SRP item that had the highest proportion of AGYW agreeing was the statement “He wants to know where I am all the time” (57.2%), followed by “He has more to say than I do about important decisions that affect us” (31.8%), and “When I wear things to make me look beautiful he thinks I may be trying to attract other men” (27.6%).

Among the AGYW survey participants who were identified as being at risk of HIV infection (had sex within 12 months before the survey and did not identify as HIV-positive), only 22.3% reported that they used male or female condoms 90–100% of the time with their last partner. As shown in Table 3, of the reasons why AGYW did not use condoms 100% of the time when they had sex, 2.6% were worried about what their partners would think if they asked to use condoms, with higher reporting in the 15–19 years age group (3.4%), than in the 20–24 years age group (1.4%). Of all AGYW respondents, 11.5% reported that they did not manage to use condoms 100% of the time because their sexual partner did not want them to use condoms, again with higher reporting amongst younger girls (15.2%), as compared to older girls (9.8%). There were also differences in reporting between provinces, with the highest reporting of not using condoms due to concern over what a partner would think in the Free State (6.3%), and highest reporting of not using a condom due to a partner not wanting to in KwaZulu Natal (16.9%).

**Table 2:** Sexual Relationship Power Scale Reporting and percentage of time used condoms with last partner (N=381)

	Used condoms 0-49% of the time (N=126)	Used condoms 50-75% of the time (N=128)	Used condoms 76-100% of the time (N=127)	Total
	Freq (%)	Freq (%)	Freq (%)	Freq (%)
***SRP scale item 1: When he wants sex he expects me to agree***				
Agree	56 (34.6)	51 (31.5)	55 (34.0)	162 (42.9)
Disagree	69 (31.9)	76 (35.2)	71 (31.9)	216 (57.1)
total	125 (33.1)	127 (33.6)	126 (33.3)	
***SRP scale item 2: If I asked him to use a condom, he would get angry***				
Agree	29 (54.7)	16 (30.2)	8 (15.1)	53 (14.0)
Disagree	96 (29.5)	111 (34.1)	119 (36.5)	326 (86.0)
total	125 (33.1)	127 (33.5)	127 (33.5)	
***SRP scale item 3: He won’t let me wear certain things***				
Agree	34 (35.1)	26 (26.8)	37 (38.1)	97 (25.5)
Disagree	92 (32.4)	102 (35.9)	90 (31.7)	284 (74.5)
total	126 (33.1)	128 (33.6)	127 (33.3)	
***SRP scale item 4: He has more to say than I do about important decisions that affect us***				
Agree	46 (38.0)	46 (38.0)	29 (24.0)	121 (31.8)
Disagree	80 (30.8)	82 (31.5)	98 (37.7)	260 (68.2)
total	126 (33.1)	128 (33.6)	127 (33.3)	
***SRP scale item 5: He tells me who I can spend time with***				
Agree	26 (38.8)	18 (26.9)	23 (34.3)	67 (17.6)
Disagree	100 (31.9)	110 (35.0)	104 (33.1)	314 (82.4)
total	126 (33.0)	128 (33.6)	127 (33.3)	
***SRP scale item 6: When I wear things to make me look beautiful he thinks I may be trying to attract other men***				
Agree	36 (34.3)	41 (39.0)	28 (26.7)	105 (27.6)
Disagree	89 (32.4)	87 (31.6)	99 (36.0)	275 (72.4)
total	125 (32.9)	128 (33.7)	127 (33.4)	
***SRP scale item 7: He wants to know where I am all of the time***				
Agree	75 (34.4)	75 (34.4)	68 (31.2)	218 (57.2)
Disagree	51 (31.3)	53 (32.5)	59 (36.2)	163 (42.8)
total	126 (33.1)	128 (33.6)	127 (33.3)	
***SRP scale item 8: He lets me know I am not the only partner he could have***				
Agree	19 (32.8)	19 (32.8)	20 (34.5)	58 (15.3)
Disagree	107 (33.2)	108 (33.5)	107 (22.2)	322 (84.7)
total	126 (33.2)	127 (33.4)	127 (33.4)	

**Table 3:** Reasons why AGYW did not use condoms 100% of the time when she had sex in last 3 months (n = 351)

	Klipfontein, Western Cape	Bojanala, North West	King Cetshwayo, KZN	Ehlanzeni, Mpumalanga	Nelson Mandela Bay, Eastern Cape	Thabo Mofutsanyana, Free State	Total			
	Freq/N (%)	Freq/N (%)	Freq/N (%)	Freq/N (%)	Freq/N (%)	Freq/N (%)	Freq/N (%)	95% CI		
**AGYW did not use condoms 100% of the time when she had sex because she was worried about what her partner would think if she asked to use condoms**										
15-19 years	0/10 (0.0)	0/20 (0.0)	1/28 (3.6)	1/17 (5.9)	1/17 (5.9)	2/20 (10.0)	5/145 (3.4)	1.0 - 8.7		
20-24 years	0/34 (0.0)	0/26 (0.0)	2/55 (3.6)	1/28 (3.6)	1/28 (3.6)	2/43 (4.7)	3/215 (1.4)	0.4 - 4.0		
Total	0/44 (0.0)	0/46 (0.0)	3/83 (3.6)	2/70 (2.9)	2/45 (4.4)	4/63 (6.3)	9/348 (2.6)	1.0 - 5.3		
**AGYW did not use condoms 100% of the time when she had sex because her sexual partner did not want her to use condoms**										
15-19 years	3/10 (30.0)	4/20 (20.0)	5/28 (17.9)	4/44 (9.1)	3/17 (17.6)	0/20 (0.0)	22/145 (15.2)	8.8 - 23.8		
20-24 years	4/34 (11.8)	1/26 (3.8)	9/55 (16.4)	2/26 (7.7)	1/28 (3.6)	4/43 (9.3)	21/215 (9.8)	5.4 - 15.7		
Total	7/44 (15.9)	5/46 (10.9)	14/83 (16.9)	6/70 (8.6)	4/45 (8.9)	4/63 (6.3)	40/347 (11.5)	8.0 - 15.8		

**Table 4:** Multivariate analysis of SRPS measures associated with percentage condom use with last sexual partner

	Percentage of time used condoms with last partner		
	**Used condoms 0-49% of the time**	**Used condoms 50-75% of the time**	**Used condoms 76-100% of the time**
	Relative risk ratio (95% CI)		
***SRP scale item 2***^*#*^: ***If I asked him to use a condom, he would get angry***			
Disagree (ref)	-	-	-
Agree	-	0.50 (0.27-0.95)*	0.21 (0.09-0.48)**
***SRP scale item 4***^*##*^: He has more to say than I do about important decisions that affect us*			
Disagree (ref)	-	-	-
Agree	-	1.03 (0.60-1.77)	**0.56 (0.31-1.00)**

*p-value<0.05; **p-value<0.01

^*#*^For SRPS_2 we adjusted for whether AGYW had ever been pregnant, HIV and relationship status, and use of contraceptives other than condoms

^*##*^For SRPS_4 we adjusted for whether AGYW had ever been pregnant, relationship status, age-disparate sexual relationships and use of contraceptives other than condoms

As shown in Table 4, after adjusting for potential confounders through an iterative series of bivariate analyses, the SRP scale items which were significantly associated with condom use outcomes were: SRP_2 (“If I asked him to use a condom, he would get angry”) and SRP_4 (“He has more to say than I do about important decisions that affect us”).

In the SRPS_2 model, participants were 79% (95% CI: 0.09–0.48) less likely to be in the highest condom use group if they agreed that if they asked their sexual partner to use a condom, he would get angry. Based on bivariate analyses, the model for SRPS_2 includes the confounders: having ever been pregnant, HIV status, relationship status, and use of contraceptives other than condoms in the past six months.

AGYW were 44% (95% CI: 0.31-1.00) less likely to be in the highest condom use group if they reported that their partner had more to say than they did about important decisions that affect them in the SRPS_4 model. The model for SRPS_4 was adjusted for having ever been pregnant, relationship status, age-disparate sexual relationships, and use of contraceptives other than condoms in the past six months.

## Qualitative Findings

**Fig. 1:**
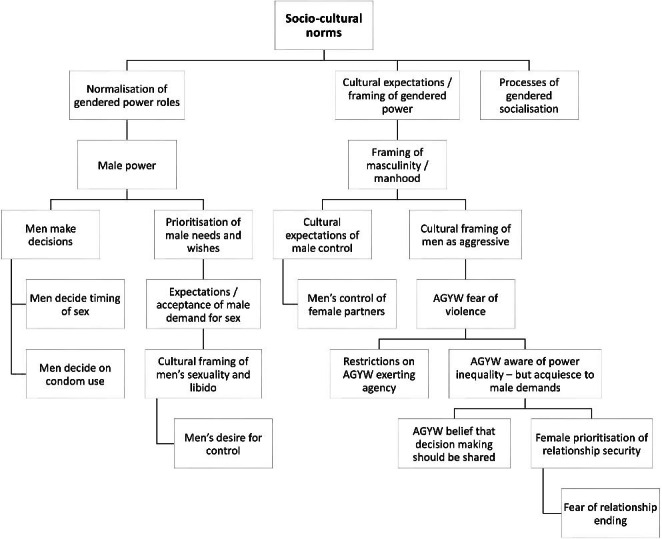
Qualitative Analysis Coding Tree

The coding tree in Fig. 1 presents the key emergent themes and subthemes in the qualitive data. Themes are further unpacked below, alongside illustrative quotations from the English transcripts. Participant details (province, sample group) are provided in brackets at the end of each quotation.

### Decision-Making Power in Relationships – Socio-Cultural Norms and Male Power

Emerging clearly from the qualitative narratives was evidence of persistent socio-cultural norms around gendered power within the relationship context. When asked to share their views on who makes decisions in sexual and romantic relationships, many of the AGYW described inequities in relationship power, voicing perspectives that generally it is the males who make decisions, following deeply embedded socio-cultural norms.

*Out of 100%, I can say 99.9% of the time, men make decisions… meaning that men rule… There is this thing (norm) that says “A man is the head of a family” …even my son knows that… That mentality is always there… men will always rule… the whole thing of saying “we are the Sothos” and everything that a man says goes... a woman always agrees… men grow up with the mentality that they are the heads of the families. There is no 50/50. (Free State, AGYW 20–24 years)*

Due to deeply entrenched socio-cultural norms and expectations relating to gendered power, AGYW explained that they avoid attempting to challenge male dominance. They refrain from exerting their own agency out of fear that disagreeing with men may be interpreted as challenging cultural norms, and in particular the hierarchical order of men ‘as the head of the family’. AGYW feel ‘scared’ of their partners and possible reprisal to any perceived acts of resisting male dominance.

*I think decision-making is supposed to be happening equally, but in my view decisions are always taken by boys, because they have the power more than us and they take advantage… that’s why decisions are taken by them… a man or boy… is regarded as the head of the family in the Xhosa culture… they use that against girls and think that it will always be their way in the relationship… most girls are shy… we listen to what they say and become their puppets… they have power because once they utter something, we agree with that even though we know it’s not right. But you have no other way because you are scared of the person you are dealing with. So, that’s why they have power. (Eastern Cape, AGYW 15–19 years)*

The perspectives of the male peer respondents corroborated these narratives relating to the strength of socio-cultural norms ascribing males with decision-making power in relationships with women. Male respondents explained the socialisation process in which these gendered norms are perpetuated through social institutions such as the church.

*The cultural practices that we follow, we have been taught… for me, when I went to church, we are taught that the man is the head of a house, he leads everything. (Eastern Cape, Male Peer)*

Male peer respondents from across the study sites, from various ethno-linguistic groups in South Africa, shared the view that male dominance and control in relationships are the norm.

*When it comes to decision making in a relationship, between a man and a woman… Uhhhh… it’s obvious, it is always the man… gents have the upper hand… When I say the upper hand I mean gents taking decisions… when coming to issues of sex decisions… obviously it is the man. (Free State, Male Peer)*

Young men’s narratives illustrated the ways in which definitions of masculinity centre around male control and decision making dominance in relationships.

*Men try to define themselves, that they are men, especially amongst Zulus or Xhosas… they don’t want equality in a relationship… they control… they don’t want to hear the opinions of their partners… in my culture I believe that men are the head of the family... I’m a Tswana man… so I believe a man must make decisions. (North West, Male Peer)*

Despite the diversity within South Africa, young men explained that the power that men have to make decisions in relationships is a culturally informed norm and expectation within most South African ethno-linguistic groups.

*We (South Africans) vary according to… our diversity, culturally… beliefs… customs… they have an impact when it comes to relationships… let’s say you’re from a Nguni tribe. Most of the Nguni people they believe that a man is the one who is the head of the family… it plays a very… very big role when it comes to a relationship… In general, men are the ones that make decisions in a family… you can’t have a household where a women is the shot caller (decision maker)… women have to have to obey what a man says … You have a main lion… a senior lion. A main lion is the king of the jungle… a woman has her own place… a man has his ways of doing things, he has roles as a man, a man is the head of the house… in every situation. (North West, Male Peer)*

Narratives from male respondents suggested that the sociocultural framing of masculinity and manhood align with ideas of male control and gendered power imbalances.

*Some men try to define themselves as men especially you know Zulus or Xhosas… they don’t want equality in a relationship. They feel that they are the ones who are the head of their families so they control… they don’t want to hear opinions of their partners. (North West, Male Peer)*

Some of the male respondents articulated their beliefs that women and girls should respect men, prioritising their male partner’s desires over their own, and considering his needs in every decision.

*Girls should respect the gents… She should always think about him, like “if I do this is he going to be happy or not?”, if he won’t be happy then she should not do it. (Free State, Male Peer)*

Some of the male respondents suggested that although they believed that equality between men and women is important in relationships, community norms were slow to change, and that in practice, men continue to hold the power.

*Young boys should be taught that everyone is equal and important. It can help them to nurture healthy relationships... in reality, in the community… men make most of the decisions in relationships… I think responsibilities should be equally shared, but in the community it is not like that. (Eastern Cape, Male Peer)*

Some of the male peer respondents believed that women should hold more decision-making power, articulating an awareness of the influence of dominant masculinities in relationship dynamics. However at the same time there was an acknowledgement of the power of cultural norms in dictating these gendered dynamics.

*It doesn’t mean that when I’m a man I have more authority than you as a woman… no it’s not like that… you can take decisions you as a woman… I should respect the fact that you’re human being and I shouldn’t let my masculinity be ahead of everything… but it depends on culture… it has an impact when it comes to relationships. (North West, Male Peer)*

One reason for men exerting decision making dominance over women, according to the views of AGYW, was that men like to control their female partners’ behaviour.

*It’s men who make decisions in relationships… at home… at the house… they want us to listen to them, they want to be in control. A woman is not supposed to say anything… Like when a woman says “my husband, I want to go somewhere”, a man refuses… because he wants to control her… So a woman is afraid to talk to a man… because he is the one who is supposed to talk. (North West, AGYW 20–24 years)*

Some of the AGYW respondents insinuated that men had double-standards, engaging in behaviours themselves that they prohibited for their partners, such as partying and drinking alcohol.

*Guys in most instances take decisions for us girls… Like I want to groove (party)… I love (drinking) beer, but he will tell you don’t go there (out drinking), but he will go where he said you should not go. (Free State, AGYW 20–24 years)*

Some of the AGYW voiced indignation at the unfairness of power inequities in traditional gendered norms, while recognising that they continue to be perpetuated and normalised.

*Men rule. Men want to rule… this is the normal thing. We normalise it and it’s wrong! (Free State, AGYW 20–24 years)*

Even amongst those AGYW who expressed the sentiment that they feel men should not have the control, and that females should also have agency within relationships, there was a sense of resignation regarding the way things are, and an acquiescence to male demands for power.

*It’s boys who normally make decisions... Because boys… they like to control too much… they feel that they are the ones who have to make decisions in a relationship, and as a girl you don’t have a say in what you do... I don’t feel good about it because we should also have a say… boys underestimate us… we don’t have a say, actually we are not supposed to oppose anything they say, we must just listen to them… to what they say. (North West, AGYW 15–19 years)*

Apparent in the narratives of AGYW, was a sense that although it is not right that men make the majority of decisions in relationships, there was a necessity in accepting that this is the way things are.

*As I’m getting older, what I have experienced, it has always been the men who make the decisions for us… basically on everything. (Eastern Cape, AGYW 15–19 years)*

Illustrating the constraints upon females exerting their agency in decision making, some AGYW explained that at times, men use violence to enforce their control over female partners.

*It’s actually men who make decisions… they make you do something that you don’t want to do. They even force you to do it… they beat you. If you don’t want to do something that he wants you to do… he will accuse you of doing something wrong. (Mpumalanga, AGYW 15–19 years)*

#### Decisions About Sex

In addition to investigating general decision making in heterosexual relationships, we asked participants about decision making specifically relating to sex. In the survey, 42.9% of AGYW reported that they feel they are expected to agree to a male partner’s demand to have sex. Amongst the qualitative respondents, the majority of AGYW shared the perspective that generally it is the male partners who decide about timing of sex. Some AGYW suggested that the idea that women could make decisions related to sex was inconceivable.

*On decisions related to sex… it’s men obviously who make those [Laughing]… there’s no way (a girl would decide that)!… [Laughing]… you tell him that you’re tired… you don’t want to (have sex)... then he will start to be angry. (North West, AGYW 20–24 years)*

In the narratives of AGYW, there were descriptions suggesting that male power in the sexual context left little space for AGYW to exercise agency and consent.

*Men rule decisions on sex… men lead. Even if you say “No, I am not ready!”, they will just manipulate you… you will agree. (Free State, AGYW 20–24 years)*

According to AGYW, men’s manipulation of female partners to agree to have sex entailed tactics such as threating to end a relationship unless sex takes place.

*It is boys who decide about sex… sometimes as a girl, you don’t want to do it. The boy will say that if I don’t want to do it, we better breakup. So, a lot of girls are forced in to doing it because they don’t want to lose their partners. (Mpumalanga, AGYW 20–24 years)*

Illustrating the predominance of sexual scripts that normalise male control and aggression in the sexual sphere, AGYW respondents described situations in which male partners enact violence, forcing their female partners to have sex.

*In our community… men are controlling. Girls are meek and abused, and the boys make the rules, girls are told what to do and what not to do... some girls are forced (to have sex), some are raped, some are hurt, and some are beaten by men, when they don’t want to (have sex). (Western Cape, AGYW 20–24 years)*

Male peer respondents also shared their perspectives on decision-making around sex in heterosexual relationships. One view expressed was that men know they can get sex elsewhere if their partner refuses. This creates situations similar to that described above, where women only agree to have sex out of fear that they will lose their partner.

*Decisions about sex? It’s obvious, it is the male… if you don’t want to have sex with him, he knows that there are others. If you don’t want to give it to him, he will go somewhere else and get it because there’s a lot of girls he can go to. So, women and young girls often have that pressure, knowing that if they don’t agree, a man can go anywhere for sex. In that way it is usually men who make that decision (to have sex). (KZN, Male Peer)*

Aligning with norms around masculinity, male sexuality and libido, and the expectation that men should want to have sex more often that their female partners, male peer respondents suggested that men often beg for sex.

*For decisions regarding sex... well [laughing] in reality we as guys usually beg for sex from the girls. (Eastern Cape, Male Peer)*

Despite the salience of narratives relating to male power and control of sex, some of the AGYW respondents articulated beliefs that decisions about sex should be made by both partners equally.

*It shouldn’t be a particular person (who decides about sex), it should be what both parties want… It shouldn’t be based on the male wanting it alone, it should be what you both want. (Western Cape, AGYW 20–24 years)*

#### Decisions About Condoms

Echoing the survey findings on condom use, AGYW respondents in the qualitative study described their perceptions of male partners being manipulative and controlling in terms of condom use as well as the timing of sex. AGYW explained that men often say that they do not want to use condoms due to discomfort.

*The condom is so good… but a lot of men will say “I don’t want to use the condom because it hurts”. (KZN, AGYW 20–24 years)*

However it was suggested that men’s complaints related to condoms being painful is merely a way of manipulating their female partners to engage in condomless sex.

*Men are manipulative… he will say “No, this thing (condom) hurts me”... Then women… if they are mentally weak, they will agree to not using it… not seeing that they are being controlled. (Free State, AGYW 20–24 years)*

Male peer respondents agreed with the view that most often, it is ultimately the male partner who decides whether condoms should be used or not.

*Guys make decisions on condoms most of the time. For me, personally… I’ve never had never had times… with my recent or past partners, that they would actually… hound me (demand) to use a condom, unless it is a decision I come to myself. (Eastern Cape, Male Peer)*

Some of the AGYW respondents agreed that while it is usually the male partner who make decisions about the timing of sex, mutual decisions can be made about condom use.

*(In sex) it’s boys (who make decisions)… (with condoms) that decision depends on an individual… you cannot depend on someone else’s say (decisions), your partner must also hear what you have to say… Don’t listen to one side, it must be 50/50. (North West, AGYW 15–19 years)*

AGYW expressed the sentiment that although women ideally should have a right to demand condom use, male partners may not listen to that demand. In the end it is men who have the final say regarding whether condoms are used or not.

*On the decision on when to have sex and when to use of condom… It is him that decides, but if you are against that, you have a right to say what you think, and he should listen to you. (KZN, AGYW 20–24 years)*

Contrary to these views above, some AGYW respondents suggested some women yield more power in demanding that their partner use a condom.

*(About the use of a condom)... the woman decides… But if the guy refuses, it’s an issue that can be discussed. Mostly it’s the woman who will say “put your condom on”. (Free State, AGYW 20–24 years)*

At times, AGYW themselves are the ones who prefer condomless sex. However, as demonstrated in the quotation below, couples are not always consistent in their condom use, or decision-making around condom use.

*I sometimes take a decision that we should use a condom… and sometimes I can tell that she is upset. But sometimes she is the one who says we’re going to use a condom… sometimes she says she doesn’t enjoy sex when we use a condom. (KZN, Male Peer)*

Some respondents felt that since decisions about sex, contraceptives and condoms affect both partners, decision-making should be shared.

*Both people should make decisions… when you get pregnant, it means you are both pregnant, so when you both take a decision (about using condoms/contraceptives), it will affect both of you. (KZN, AGYW 20–24 years)*

#### Shifting Norms

Illustrating the shifts and changes in norms around gendered power, both male and female respondents articulated views that decision-making in relationships should be equal, and that communication should be open and honest.

*I believe that we are a changing community right now, we are evolving as people and we are becoming more open with each other… decisions are best made together… if one can make a decision over the other one… that means you guys are not transparent with each other, so transparency is the most important thing you can ever have in a relationship. (North West, Male Peer)*

Supporting this view, some AGYW felt that decisions in a relationship should ideally be made through discussion and consensus, particularly when decisions affect both partners.

*In my opinion when you’re in a relationship decision making is for both of you… if it involves both of you, it’s not for one partner to make a decision, because a relationship is for two people and not one. (Free State, AGYW 20–24 years)*

Some AGYW suggested that despite gendered norms endowing men with decision-making dominance, women needed to be strong and not subservient to men.

*Usually it is the men who make decisions, but you should both come to an agreement before it is done, and don’t allow him to use his power or want to show you that he has power over you… You should both discuss. (KZN, AGYW 20–24 years)*

Some of the AGYW expressed their indignation at the power inequities, believing that women need to demand gender equality, and not accept men’s assertion of power.

*I am his equal. Men should respect, understand… Because if they don’t do that… they are not worth it… 50/50 it’s a balanced thing. (Mpumalanga, AGYW 15–19 years)*

## Discussion

Our findings suggest that despite shifting social norms, gendered power inequities remain deeply entrenched in the sexual power dynamics in heterosexual relationships amongst adolescents and young people in South Africa. Findings from both the survey and the interviews suggest that female acquiescence to male sexual demands remains a normative expectation. The power that AGYW have in their sexual relationships determines their ability to use condoms, and this in turn is affected by the relationship type. After adjusting for potential confounders through an iterative series of bivariate analyses, only two of the SRP scale items were significantly associated with condom use outcomes: item 2 (“If I asked him to use a condom, he would get angry”) and item 4 (“He has more to say than I do about important decisions that affect us”). AGYW who agreed with these statements were less likely to report high condom use. Qualitative narratives on condom use echoed the survey findings, with AGYW describing male partners’ manipulative and controlling behaviour in terms of condom use as well as the timing of sex. Both AGYW and male peer respondents in the qualitive interviews stated that it is generally the male partners who decide on timing of sex. These narratives overlap with the survey data showing that almost half of the AGYW survey participants reported that when their partner wants sex, they are expected to agree. In qualitative interviews, respondents spoke of male control over their female partners, with men’s power and dominance extending beyond the boundaries of the sexual domain. Narratives of male power and control over female partners’ behaviour were echoed in the survey data.

Prior evidence is mixed regarding the association between SRP equity and condom use. Our analysis demonstrated a significant association between two of the SRP scale items: and condom use, with AGYW agreeing with these statements significantly less likely to report high condom use. It has been suggested that one limitation in the SRP scale design is that it asks about power dynamics in primary relationships but fails to capture the nuances in different types of relationships and partners, in which condom decision making is likely to differ [8]. Despite the lack of a clear association between SRP equity and condom use, evidence does suggest an association between women’s HIV infection and low reported SRP equity [14]. Barriers to AGYW exerting agency and control over condom use, such as gendered power inequities and hegemonic masculinities, are critical mediating factors associated with heightened HIV transmission risk for AGYW [18]. Our findings bolster the existing literature describing the way in which prevalent inequitable gendered power dynamics in South African heterosexual relationships continue to constrain the sexual agency of AGYW, discouraging AGYW from taking control of their own sexual interests and sexual health [10, 19]. Additional factors that constrain AGYW sexual agency and power include age disparity and/or socioeconomic disparity between AGYW and their partners [10, 20].

Qualitative narratives from both AGYW and male peer respondents highlighted deeply embedded socio-cultural norms relating to gendered power and decision-making power in relationships between men and women. Respondents described the socialisation processes in which these gendered norms are perpetuated. Findings from the survey suggested male power over women in relationships resides in particular domains, with men exerting control over female partners’ behaviour. As seen in the narratives of male and female respondents in our study, boys are socialised into roles determining their position as heads of their households. Prior literature describes Zulu cultural norms with women assuming subordinate positions to male heads of households [21], and our study indicated that these hegemonic masculinities are present across ethno-linguistic groups included in this study (Zulu, Xhosa, Tswana and Sotho). The persistence of social norms that sustain inequitable gendered power dynamics demonstrates how resistant they are to change, and the ways in which they continue to be reproduced [22]. However, despite the expectation for South African women to accept male domination and control, gendered power norms are undergoing shifts and are being challenged, as articulated in the qualitative narratives of young men and women in our study [21].

Even though many of the AGYW in our study expressed an awareness of their rights and a belief that there should be equality in decision making in relationships, they voiced a sense of resignation to the status quo, and an acquiescence to male dominance. AGYW spoke about the way in which women refrain from enacting sexual agency out of fear of the consequences. This is further supported by our quantitative finding that AGYW who were afraid that their male partners would get angry if they asked him to use condoms, were less likely to consistently use condoms. Some AGYW expressed indignation at the power inequities, believing that decision making power should be equally shared. However at the same time, AGYW explained that exercising agency is not always possible, as men use violence to enforce their control over female partners. There is a wealth of literature describing norms around masculinity and power across African cultures; more recently research has focused on the association between traditional gender roles and violence [21–24]. The normalisation of male control, dominance, aggression and even violence in the sexual sphere was described by AGYW respondents in our study. As seen in the narratives of AGYW we interviewed, and in the broader literature from South Africa, male dominance in decision-making in the dyadic context, and the use of violence to enforce this dominance, is informed and perpetuated by hegemonic masculinities throughout South African society and across ethno-linguistic groups [25–27]. Expectations of male sexuality as exerting strength and control over women frames young men’s sexual socialisation [27]. These prevalent masculinities can be seen as consensual ideologies, which justify and sustain gendered power inequalities [28].

Some of the male respondents in our study expressed views that equality between men and women is important in relationships, but that community norms were slow to change, and that in practice, men continue to hold the power. Sexual scripts relating to gendered power in heterosexual relationships are embedded in and informed by socio-cultural norms and expectations [18]. Although socio-cultural norms are not static and unchanging, as suggested by respondents in our study, and in other South African studies, they are slow to shift [29]. In addition, the narratives of AGYW in our study who expressed a belief that gendered power in decision-making should be equal, but that it was not always possible to enact this equality, demonstrate that there can also be a duality, with persistent traditional values and expectations co-existing alongside more modern gender norms, as young people navigate shifts and changes in the socio-cultural landscape [30]. This ties in with the notion that while male power is pervasive and enduring, it is simultaneously contested and negotiated, affording women and girls some level of agency [31]. It is too simplistic to frame relationship power in the binaries of domination and subordination; instead, there is a need to examine the agency that women and girls enact in the dyadic context of heterosexual relationships [31]. The perpetuation of ‘gender inequity norms’ requires adherence to socio-culturally embedded norms on gender and sexual roles [32]. However, as seen in our study, and elsewhere, some South African adolescents and young people are demonstrating a desire to challenge traditional patriarchal practices and inequitable gender roles, signalling a potential for change [21].

The concept of “empowerment” has been central to efforts to address gendered inequalities and power imbalances, thereby accelerating development and improving the health and well-being of women and girls [33]. Empowerment is framed as a process of enhancing an individual’s capacity to exercise choice, make decisions, and critically, the ability to act on those decisions, and achieving their choice [33, 34]. Interventions that aim to “empower” AGYW are designed to increase self-efficacy and agency, and therefore improve sexual and reproductive decision-making and related health outcomes [34]. Since socio-cultural gendered norms and expectations are a key obstacle to South African AGYW’s exercise of agency in the domain of sexual and reproductive health, it is critical that interventions and programmes that aim to improve AGYW SRH empowerment address these gendered power inequalities by challenging gender norms and hegemonic masculinities of dominance [33]. There has been some success with ‘gender-transformative’ empowerment interventions in shifting harmful gender norms and roles through community level programmes; however for these interventions to be successful, it is critical to engage both young males and females in critiquing existing ideas of manhood and womanhood, the persistence of gender power inequities, and encourage more positive forms of masculinity and equal power dynamics in relationships, and fostering gender-equitable beliefs [18, 19, 27, 35].

Limitations to this study include the following: (1) We did not conduct the survey, inclusive of the SRPS and condom use questions, with adolescent boys and young men, and therefore are unable to compare reporting between genders. (2) The final survey sample realization was lower than expected. The success of the sampling strategy was dependent on AGYW being contactable by phone, therefore those who are not contactable by phone are likely to be different to, and possibly more vulnerable than those who have access to working phones, which is likely to have introduced a bias in the study findings. The survey sample comprised intervention beneficiaries and therefore might not be representative of the general AGYW population in sampled districts. (3) Limitations of the qualitative interviews relate to the sampling bias for the limited sample of male participants, who were referred to the study team by their female partners, and are therefore potentially more likely to be in more gender equitable relationships. (4) Additionally, in interviews there is always the potential for social desirability bias. However, there were several advantages to the remote data collection method, including the potential for increased disclosure of sensitive or socially undesirable behaviour (reduced social desirability bias). Important to note is that the findings in this paper relating to condom use refer mostly to male condoms. In the survey, AGYW were asked to report on condom use, inclusive of male and female condoms. Therefore the outcome measure did not specify whether the participant used male or female condoms. It is likely that very few participants used female condoms given that so few knew what a female condom was, and few had ever used one. In most cases, in the qualitative interviews, respondents did not differentiate between male and female condoms.

### Conclusions and Implications

Our findings build upon evidence showing that South African gendered power expectations and sexual scripts, shaped by deeply entrenched and dominant hegemonic masculinities, continue to be unequal, supporting the expectation of male control over women and women’s acquiesce, especially regarding sexual behaviour, and thus placing women and girls at a disadvantage [10, 14, 25, 29]. Whilst adolescents and young people in South Africa move away from some traditional cultural expectations, hegemonic masculinities in South Africa continue to legitimise men’s power over women, which manifests in decision-making power in the relationship context [3]. Our findings show a link between sexual relationship power and condom use, and illustrate that AGYW’s ability to exert agency in decision-making relating to timing of sex and condom use are influenced by dyadic-level factors including relationship power inequity, as well as socio-cultural level norms of hegemonic masculinity. These findings corroborate the assertion that South African AGYW’s sexual and reproductive health and agency in the sexual space is highly influenced by deeply entrenched and persistent hegemonic norms of masculinity and unequal gendered power dynamics in heterosexual sexual scripts [18, 36].

The findings of this survey related to sexual relationship power imply the need for interventions to empower AGYW, as well as the need to expand interventions for adolescent boys and young men, and male sexual partners. Programmes need to include components that work to address young people’s attitudes towards interpersonal gender power dynamics, and support equitable sexual decision making [10]. However, efforts to implement empowerment programming aimed at increasing AGYW’s self-efficacy for condom use and sexual negotiation must consider the way in which dyadic level sexual scripts and relationship dynamics, including unequal power, are likely to constrain AGYW’s enactment of their sexual agency [18]. Given the influence of socio-cultural level norms and hegemonic masculinities in South Africa, further attention needs to be paid to community level intervention approaches that aim to create conducive environments that enable AGYW to exert control in sexual negotiations by addressing harmful social gender norms that perpetuate inequalities [10, 18].

Gender-transformative interventions that aim to address problematic gender norms, working with both males and females, are a critical part of addressing power inequities [10, 34]. Strategies might include the promotion of healthy and respectful relationships, and role modelling of gender equitable relationships by female and male co-facilitators [10]. Such gender-transformative interventions could be built into school-based programming and curricula [36]. In addition, the broader context needs to be considered through community-level interventions, which are key in creating an enabling environment for shifting problematic gender norms and restrictive masculinities, addressing gender inequalities that exist at the structural level [26, 30, 34, 37].

**Declarations**.

## Data Availability

The datasets used and/or analysed during the current study are available from the corresponding author on reasonable request.

## Footnotes

1 https://www.samrc.ac.za/intramural-research-units/healthsystems-herstory.

2 More details on the survey methodologies can be found at https://www.samrc.ac.za/intramural-research-units/healthsystems-herstory.
